# NEW SPINS ON CLASSIC IDEAS ABOUT CONTEXT IN ADULT EMOTIONAL DEVELOPMENT

**DOI:** 10.1093/geroni/igac059.1440

**Published:** 2022-12-20

**Authors:** Tabea Springstein, Tammy English

## Abstract

Individuals often experience improvements in emotional well-being into old age. Understanding mechanisms contributing to these emotional outcomes in daily contexts can inform ways to support healthy aging. Development is embedded within various contexts that shape individuals’ experiences. Novel perspectives are emerging on how to conceptualize context and the way it can contribute to emotional development during the aging process. This symposium illustrates four innovative ways to consider contextual contributions to emotional well-being across adulthood. The first talk will use experience sampling to illustrate age differences in how daily situations contribute to emotion regulation related processes, showing that older adults can more easily distinguish between emotions when in familiar situations. The second talk will take a fresh perspective on psychosocial contexts by distinguishing between types of social interactions in couples, highlighting the important role of affection for well-being in adulthood. The third talk will introduce the idea that the body itself provides context for emotional processes, showcasing that the way this context affects emotional experience changes as individuals age. The fourth talk will center on how renewing our classical developmental models of context in modern ways can help to overcome shortcomings of previous research and provide insight into how engagement with environmental features contributes to well-being across the lifespan. In sum, this symposium features innovative perspectives on how context can be leveraged to gain a deeper understanding of psychosocial development into old adulthood and illustrates specific ways individuals can navigate their social world to preserve or improve mental health across adulthood.

